# Supplementary material for: Transient frontal spectral events from EEG predict antidepressant response to sertraline in depression
Source: J Psychiatr Res. Author manuscript; Available in PMC 2026 May 29. (PMC13221100; doi:10.1016/j.jpsychires.2026.03.010)
Supplement: Supplementary Material [file NIHMS2165432-supplement-Supplementary_Material.docx]

**Supplementary Information**

*Determination of Factor-of-Median (FOM) threshold cutoff*

*
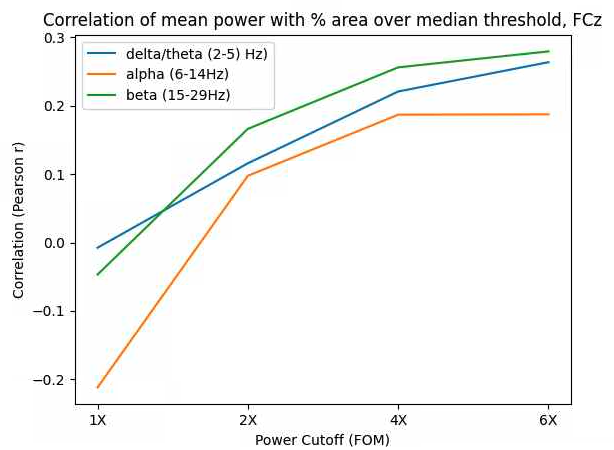
*

***Supplementary Figure 1.*** *Correlation between the power in the area above cutoff (that is, frequencies and time points that would be counted as transient events) in the frequency x time spectrogram and mean power in the frequency bands of interest. 6 x FOM had the highest correlation with mean power in all frequency bands, validating this choice of cutoff in the current study.*

*Model training, validation, and testing*

*
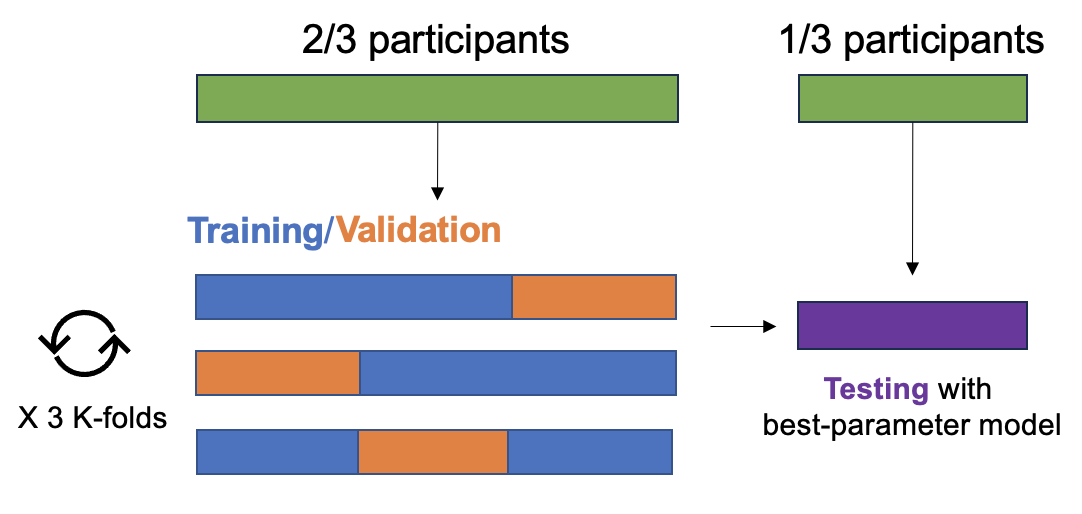
*

***Supplementary Figure 2.*** *Diagram of model training/validation and testing procedures. The cross-validated grid search and training/validation procedure was performed on 2/3 of participants using 3 K-folds for cross-validation. Testing was performed on the held-out 1/3 of participants using the resulting model with the best hyperparameters.*

*Range of SEF in Sertraline Group*

*
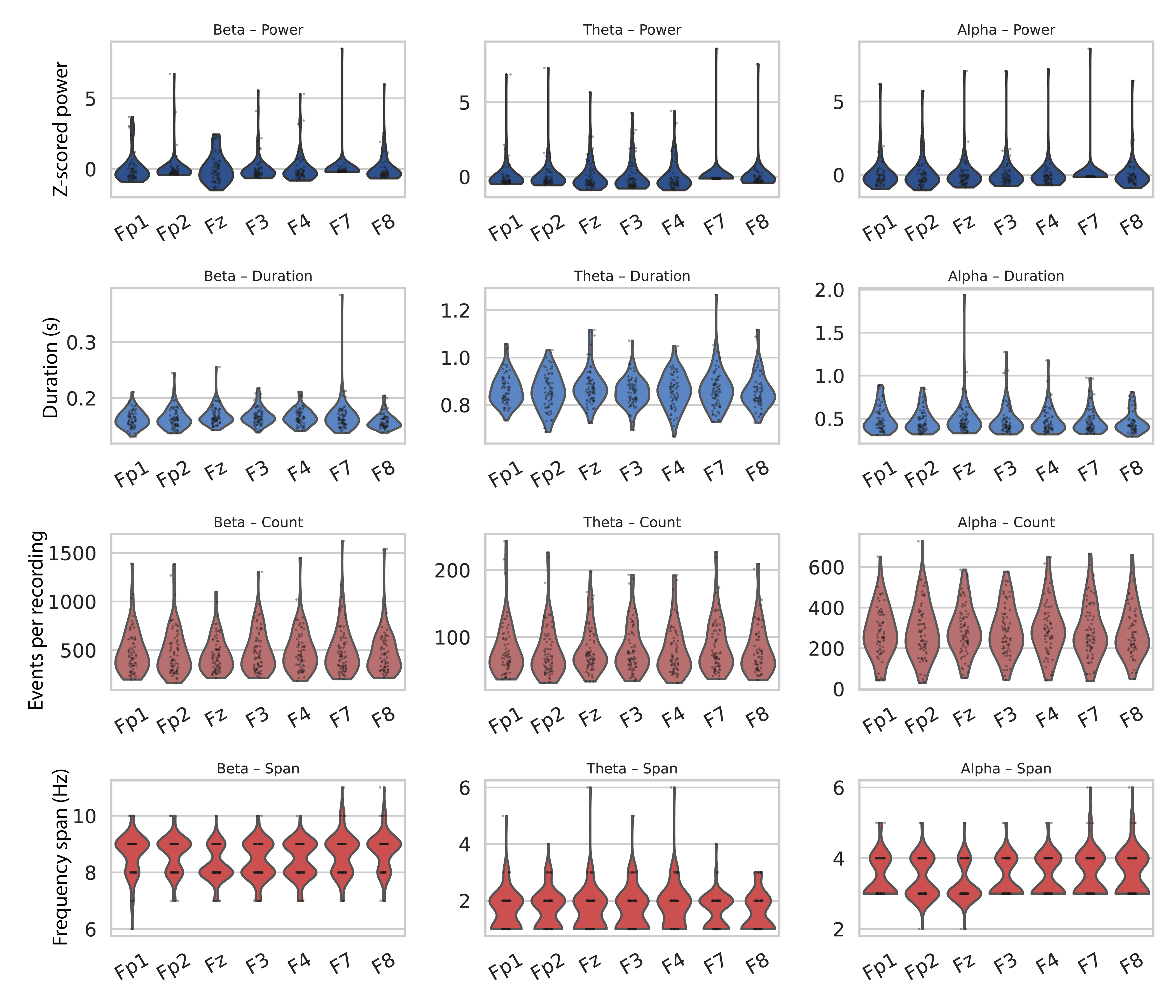
*

***Supplementary Figure 3.*** *Range of SEF at each channel and band for the subsample (N = 82) who received sertraline treatment.*

*Models cannot significantly classify binary sertraline treatment response/nonresponse with SEF only, APF, or SEF+APF*

As described in the Methods and Results section, classification models predicting categorical (responder vs. non-responder) treatment outcomes from baseline SEF did not perform significantly above chance. For completeness and to compare performance against models presented in the literature, we also tested the performance of classification models constructed with Average Power Features only and with both SEF and APF. None of the classification models tested performed above chance at predicting sertraline responder status in the EMBARC dataset (see **Supplementary Table 1**).

| *Features included* | *Model type* | *Predicted variable* | *Accuracy (F1): Training/Testing* | *P value: Training/Testing* |
| --- | --- | --- | --- | --- |
| APF only^1^ | KNN | Response (y/n) | .64 | - |
| APF only^2^ | SVM | Response (y/n) | .79 | - |
| APF only^3^ | RF | Response (y/n) | .88 | - |
| SEF only | SVM | Response (y/n) | .54/.58 | .27/.17 |
| APF | SVM | Response (y/n) | .54/.35 | .29/.70 |
| APF+SEF | SVM | Response (y/n) | .49/.60 | .53/.14 |
| SEF only | KNN | Response (y/n) | .02/.42 | .64/.45 |
| APF | KNN | Response (y/n) | .60/.39 | .09/.78 |
| APF+SEF | KNN | Response (y/n) | .59/.38 | .07/.56 |
| SEF only | RF | Response (y/n) | .93/.94 | .37/.22 |
| APF | RF | Response (y/n) | .52/.61 | .38/.13 |
| APF+SEF | RF | Response (y/n) | .45/.22 | .68/.95 |

***Supplementary Table 1.*** *Accuracy and significance of published models of binary classification models of antidepressant response and classification models tested in the current study. 1: Schwartzmann et al., 2023; 2: Zhdanov et al., 2020; 3: Jaworska et al., 2019.*

*Support Vector Regression does not significantly predict treatment response with the non-linear radial basis kernel with SEF, APF, or both.*

To assess whether models that implement non-linear combinations of features instead (as opposed to linear combinations in our EN model) 1) significantly predict %change in HAM-D from SEF and/or 2) have performance improved by the inclusion of SEF, we constructed and tested the performance of Support Vector Regression (SVR) models using the radial basis kernel. SVR did not perform above chance at predicting %change HAM-D with APF, or a combination of both SEF and APF. However, SVR with SEF alone yielded a significantly predictive training-set model, though the testing-set model did not quite reach significance. This could indicate potential utility of non-linear combinations of SEF in predicting treatment response.

| *Features included* | *Model type* | *Predicted variable* | *NRMSE: Training/Testing* | *P value: Training/Testing* |
| --- | --- | --- | --- | --- |
| SEF only | SVR | %change HAM-D | -38.10/-34.44 | .02/.06 |
| APF | SVR | %change HAM-D | -40.20/-39.00 | .35/.74 |
| APF+SEF | SVR | %change HAM-D | -0.50/-0.52 | .14/.72 |

***Supplementary Table 2.*** *Accuracy and significance of a non-linear model of %change HAM-D (i.e., support vector regression with the radial basis kernel) tested in the current study.*

*Sensitivity of model outcomes to factor-of-median SEF detection threshold*

To test the effect of chosen factor-of-median detection threshold used to quantify spectral events in the SpectralEvents toolbox, we tested model performance when using SEF derived from events quantified at 4 x and 2 x factor-of-median power. We found that models including SEF quantified at these thresholds did not successfully generalize to predict above-chance in the validation stage.

| *Features included* | *Model type* | *Predicted variable* | *NRMSE: Training/Testing* | *P value: Training/Testing* |
| --- | --- | --- | --- | --- |
| SEF only (6xFOM) | EN | %change HAM-D | -37.34/-32.03 | .005/.03 |
| SEF only (4xFOM) | EN | %change HAM-D | -37.17/-35.68 | .004/.13 |
| SEF only (2xFOM) | EN | %change HAM-D | -36.76/-40.77 | .002/.40 |

***Supplementary Table 3.*** *Accuracy and significance of elastic net models with SEF extracted using different factor-of-median detection thresholds.*

*Frontopolar beta duration does not differ between pre-treatment baseline and 1 week on sertraline.*

While baseline Fp1 duration is predictive of post-treatment symptom changes after eight weeks of treatment, we did not see a significant change in beta duration one week into treatment (t = -1.27, p = .22), which is the only other timepoint at which EEG was collected in this study.

**
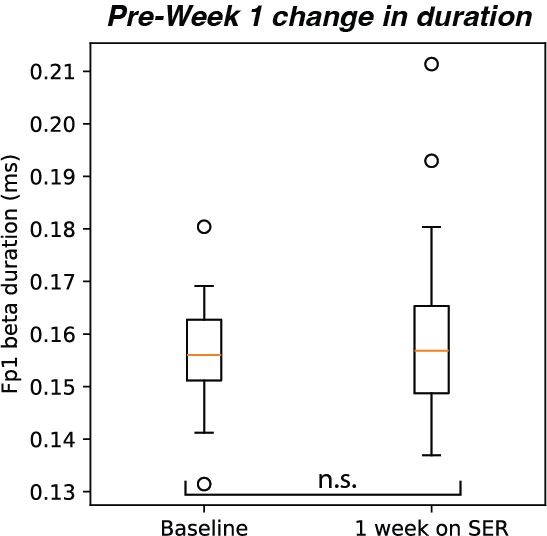
**

***Supplementary Figure 4.*** *Fp1 beta duration does not significantly change during one week of sertraline treatment. * = p < .05; n.s. = not significant.*
